# Cost‐effectiveness of screening and treatment using direct‐acting antivirals for chronic Hepatitis C virus in a primary care setting in Karachi, Pakistan

**DOI:** 10.1111/jvh.13422

**Published:** 2020-11-04

**Authors:** Nyashadzaishe Mafirakureva, Aaron G. Lim, Gul Ghuttai Khalid, Khawar Aslam, Linda Campbell, Hassaan Zahid, Rafael Van den Bergh, Gregoire Falq, Camille Fortas, Yves Wailly, Rosa Auat, Dmytro Donchuk, Anne Loarec, Joanna Coast, Peter Vickerman, Josephine G. Walker

**Affiliations:** ^1^ Population Health Sciences Bristol Medical School University of Bristol Bristol UK; ^2^ Operational Center Brussels Médecins Sans Frontières Brussels Belgium; ^3^ Operational Center Brussels Médecins Sans Frontières Islamabad Pakistan; ^4^ Epicentre, Paris France; ^5^ NIHR Health Protection Research Unit in Behavioural Science and Evaluation University of Bristol Bristol UK

**Keywords:** chronic hepatitis C, cost‐effectiveness, low‐income population, Pakistan, treatment costs

## Abstract

Despite the availability of effective direct‐acting antiviral (DAA) treatments for Hepatitis C virus (HCV) infection, many people remain undiagnosed and untreated. We assessed the cost‐effectiveness of a Médecins Sans Frontières (MSF) HCV screening and treatment programme within a primary health clinic in Karachi, Pakistan. A health state transition Markov model was developed to estimate the cost‐effectiveness of the MSF programme. Programme cost and outcome data were analysed retrospectively. The incremental cost‐effectiveness ratio (ICER) was calculated in terms of incremental cost (2016 US$) per disability‐adjusted life year (DALY) averted from the provider's perspective over a lifetime horizon. The robustness of the model was evaluated using deterministic and probabilistic sensitivity analyses (PSA). The ICER for implementing testing and treatment compared to no programme was US$450/DALY averted, with 100% of PSA runs falling below the per capita Gross Domestic Product threshold for cost‐effective interventions for Pakistan (US$1,422). The ICER increased to US$532/DALY averted assuming national HCV seroprevalence (5.5% versus 33% observed in the intervention). If the cost of liver disease care was included (adapted from resource use data from Cambodia which has similar GDP to Pakistan), the ICER dropped to US$148/DALY, while it became cost‐saving if a recently negotiated reduced drug cost of $75/treatment course was assumed (versus $282 in base‐case) in addition to cost of liver disease care. In conclusion, screening and DAA treatment for HCV infection are expected to be highly cost‐effective in Pakistan, supporting the expansion of similar screening and treatment programmes across Pakistan.

## 1 INTRODUCTION

An estimated 71 million people world‐wide are chronically infected with Hepatitis C virus (HCV), leading to 400 000 annual HCV‐related deaths.^1^ Eighty per cent of HCV infections are in low‐ and middle‐income countries (LMIC),^2^ with Pakistan harbouring the second‐largest HCV‐burden in the world (7.5 million infected persons in 2015).^3^ Without treatment scale‐up, the number of infected persons in Pakistan is projected to rise to 12.6 million by 2030.^4^ Furthermore, in Pakistan the HCV epidemic is generalized, with most HCV transmission attributable to community and medical practices.^5^, ^6^

Direct‐acting antivirals (DAAs), available globally since 2014, offer high cure rates (sustained virologic response (SVR) over 90%) with few side effects for all HCV‐infected patients.^7^, ^8^ However, access in most LMICs has been limited by their high cost,^9^, ^10^ the lack of infrastructure required for scaling up treatment, and the need for complex diagnostics and monitoring during treatment.^11^

Pakistan has had national hepatitis prevention and control programmes in place since 2005.^12^ Despite this, most HCV treatment programmes in Pakistan have been offered through specialized private or government hospitals with limited access and high out‐of‐pocket expenses for most patients.^13^, ^14^ As of 2015, approximately 150,000 patients were treated annually. However, to reach global HCV elimination targets in Pakistan, the number of treatments needs to be scaled up to at least 500 000/year,^4^ which will require an expansion of testing and treatment beyond specialized hospitals.

Médecins Sans Frontières (MSF), in collaboration with local organization SINA Health Education and Welfare Trust, operated a primary health clinic in Machar Colony, an informal settlement in Karachi with a high prevalence of risk factors for Hepatitis C (6%).^14^, ^15^ MSF is an international, independent medical humanitarian organization that provides medical assistance or care to people affected by conflict, epidemics, disasters or exclusion from healthcare. Patients who come to the MSF clinic are treated with no charge to the patients. In 2015, the MSF clinic in Machar colony started offering screening for HCV and all‐oral (interferon‐free) DAA treatment free of charge to their patients.

Although numerous studies in Pakistan have documented favourable clinical outcomes for DAA‐based treatment,^15^, ^16^, ^17^, ^18^ there is a paucity of information on their costs and cost‐effectiveness. This information would help inform decision‐making related to programme planning and resource allocation for expanding HCV treatment access.^13^ Previous economic evaluations of DAA‐based HCV treatment programmes in LMICs have generally not used locally derived cost or programme outcome data^19^, ^20^, ^21^ and have not considered the provision of screening and treatment in a primary health clinic.^22^, ^23^

In this study, we aimed to provide information to decision makers on the cost‐effectiveness of HCV screening and treatment in a setting where access to healthcare is limited. To do this, we used ‘real‐world’ cost and outcome data collected from the MSF Machar colony clinic to estimate the costs and cost‐effectiveness of testing and treating individuals with chronic HCV in a low‐income, urban primary health care setting in comparison to no screening and treatment.

## 2 METHODS

### 2.1 Study design

We assessed the cost‐effectiveness of the MSF HCV screening and DAA‐based treatment programme offered at a free of charge primary health clinic in comparison to no screening and treatment from the provider's perspective. Due to generally limited access to any healthcare in this setting,^14^ we assumed there was no access to screening and treatment for HCV in this population before the introduction of the programme.

### 2.2 Setting and model of treatment

Patient characteristics and resource utilization data were collected from the on‐going HCV screening and treatment programme, integrated into the MSF primary health clinic in Machar Colony, Karachi, Pakistan. The programme, procedures and preliminary outcomes have been described previously.^14^, ^15^ In short, the HCV programme was included as a new component to the MSF clinic in February 2015, with dedicated budget, space and personnel, although some resources were shared with existing services. All patients 18 years of age or above presenting with HCV risk factors ^14^ (Supplementary Figure S1) were referred from the outpatient department by the consulting medical officer to the in‐house MSF laboratory, where they were screened for HCV antibodies, initially using an OraQuick® rapid diagnostic test (RDT) (OraSure Technologies) and later (starting 2018) using SD Bioline RDT (Standard Diagnostic Inc, Korea). All HCV antibody positive patients were tested for HCV RNA (to confirm chronic infection) using qualitative polymerase chain reaction (PCR) testing; initially done externally, but later using quantitative PCR performed in‐house on the GeneXpert® platform (GeneXpert 1V system, Cepheid, USA). Patients confirmed to have chronic HCV infection, as well as those previously diagnosed by another provider, were evaluated and prioritized for treatment based on liver disease stage, assessed using the AST/Platelet Ratio index (APRI) score.^24^ An APRI score ≥ 1.0 (~Metavir stage F3^25^) was used as the prioritization threshold for treatment until October 2016 when it was reduced to >0.5. The medical team (two HCV doctors and one nurse) performed eligibility, baseline, on‐treatment and post‐treatment clinical evaluations for all diagnosed patients. Individual counselling sessions were given at each step of the care cascade (Supplementary Figure S1). The main regimen used was sofosbuvir with daclatasvir for 12 or 24 weeks, with or without ribavirin; sofosbuvir and daclatasvir were procured by MSF outside of Pakistan.^14^ Genotyping was performed in patients with evidence of cirrhosis to determine the length of treatment; genotype 3 patients with cirrhosis were treated for 24 weeks. Supplementary Table S3 shows the distribution of patients by treatment regimen. Sustained virological response (SVR12) was determined by PCR test 12 weeks after treatment completion.

### 2.3 Model framework for economic evaluation

**Figure 1:**
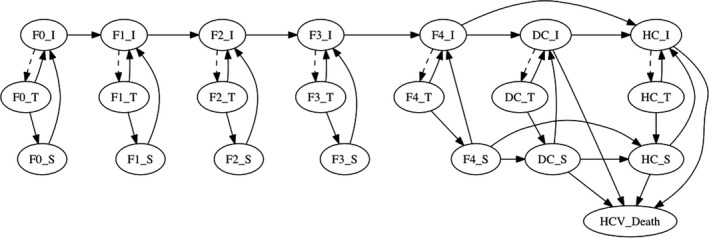
Schematic of Markov model showing how patients progress through infection and liver disease states. I, infected; T, on treatment; S, susceptible; F0, no fibrosis; F1, portal fibrosis without septa; F2, portal fibrosis with few septa; F3, numerous septa without cirrhosis; F4, compensated cirrhosis; DC, decompensated cirrhosis; HCC, hepatocellular carcinoma. Baseline mortality occurs according to cohort age (not shown in figure)

The costs and outcomes of the screening and treatment programme compared to no programme were projected over the lifetime of the patient cohort using a Markov model with an annual time step, with treatment assumed to occur in the first year of the model. The model (Figure 1) includes progression through different stages of liver disease including two mild chronic HCV states [Metavir stage F0‐F1], two moderate chronic states [Metavir stage F2‐F3], compensated cirrhosis [Metavir stage F4], decompensated cirrhosis (DC) and hepatocellular carcinoma (HCC). These seven health states have three possible sub‐states (infected, infected on treatment and recovered/susceptible). Liver‐related mortality, assumed to occur in the DC and HCC states, and age‐related mortality were modelled as absorbing health states. The number of liver transplants performed in Pakistan is unknown and assumed to be negligible, so was not modelled. Outcomes and costs (in scenarios where on‐going costs are included, see sensitivity analysis section) were discounted at a rate of 3% per year.^26^, ^27^ The model was coded in R version 3.6.1 using the package *heemod*.^28^, ^29^

### 2.4 Model inputs

#### 2.4.1 Patient characteristics and treatment outcomes

The baseline patient characteristics and distribution across different liver disease stages (mapped from the mean APRI score recorded for each patient ^25^) were based on data from HCV‐diagnosed patients following screening, with all patients in the model cohort starting in the ‘infected’ states. Patients with signs of DC or HCC were not treated by the programme, but instead were referred to a tertiary care facility. Hence, no patients entered the model with DC or HCC. Data on age‐specific life‐expectancy for Pakistan in 2016 were obtained from WHO.^30^

**Figure 2:**
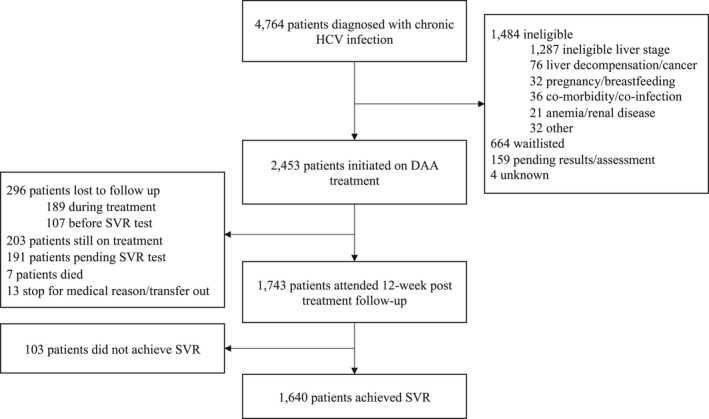
Flow chart for patients diagnosed with chronic HCV. Data available for 4764 individuals enrolled in the study as of 31 October 2018

From inception of the programme in February 2015 until 31 October 2018, 15,227 individuals attending the MSF clinic for HCV screening were tested using RDT, with an overall HCV antibody seroprevalence of 33.0% (5,025). Of these, 4,001 patients were tested for HCV RNA, with 3,127 (78.2%) diagnosed with chronic HCV infection. Over the same period, 2,279 patients came to the clinic with a positive PCR test result from elsewhere, giving a total of 5,406 diagnosed patients. Adult patients (≥18) enrolled in the study through to 31 October 2018 were extracted from the patient database on 3 May 2019. A total of 4,764 diagnosed patients were available for our analysis, of whom 2,453 (51.5%) were initiated on treatment (Figure 2).

Most diagnosed patients were female (63.9%) with a mean age of 41.3 years. Fibrosis stage was available for 4,541 (95.3%) patients, and the modelled cohort and treatment proportions are based on this group (Supplementary Table S1). All diagnosis and treatments are assumed to occur in the first modelled year. Baseline characteristics for patients in the cohort and characteristics of patients who completed treatment are provided in Supplementary Tables S2 and S3.

By 31 October 2018, of the 2,453 patients that had started treatment, 1,743 had attended their SVR12 visit, of which 1,640 were cured. The remaining patients were either still on treatment (203), awaiting their SVR12 test (191), did not complete treatment (20), or were lost to follow up (296) (Figure 2). The effectiveness of the DAA treatment programme was estimated excluding those patients that had not yet reached 12 weeks post‐treatment or were lost to follow up after successfully completing treatment, for a cure rate of 1,640/1,952 or 84.0% (95% CI 82.4‐85.6%). This cure rate was applied to all patients treated in the model.

#### 2.4.2 Disease progression rates

Health state transition probabilities were sourced from literature^31^, ^32^, ^33^ (Table 1). These transition probabilities were adjusted to reflect the higher proportion of genotype 3 in Pakistan, which has faster disease progression (see Appendix S1).^4^, ^34^ HCV treatment occurs in a proportion of infected patients in the first year of the model, and we assume disease progression and HCV‐related death does not occur in the population during this treatment initiation year. After the first year, if treatment is unsuccessful then patients return to their respective infection states and have the same risk of disease progression as untreated patients. In susceptible (cured) patients, disease progression is assumed to cease for pre‐cirrhotic patients, while for cirrhotic patients, progression continues at a slower rate compared to infected patients.^31^, ^33^ We assumed no reinfection risk in the base‐case analysis and therefore estimate impact excluding any benefits of preventing new infections due to treatment.

**Table 1:** Model parameters: baseline values, ranges and distributions

Variable	Base‐case	Distribution parameter(s)	Distribution in PSA	Source
Transition probabilities (annual)				
Mild fibrosis (F0) to Mild fibrosis (F1)	0.117	0.0051	Normal	^32^
Mild fibrosis (F1) to Moderate fibrosis (F2)	0.085	0.0036	Normal	^32^
Moderate fibrosis (F2) to Moderate fibrosis (F3)	0.121	0.0046	Normal	^32^
Moderate fibrosis (F3) to Severe fibrosis (F4)^#^	0.115	0.0041	Normal	^32^
F3 to F4 or F4 to DC hazard ratio for genotype 3	1.31	0.27, 0.033	Lognormal	^34^
F4 to DC^#^	0.039	14.6, 360.2	Beta	^57^
F4 or DC to HCC^#^	0.14	1.9, 136.1	Beta	^57^
F4 or DC to HCC hazard ratio for genotype 3	1.80	0.59,0.061	Lognormal	^34^
F4 to DC hazard ratio after SVR achieved	0.07	‐1.5, 0.21	Lognormal	^31^
F4 or DC to HCC hazard ratio after SVR achieved	0.23	‐2.7, 0.48	Lognormal	^33^
DCC to liver death	0.130	0.11, 0.15	Uniform	^57^
HCC to liver death	0.43	0.37, 0.49	Uniform	^57^
HCV reinfection	0	0, 0, 0.037	Triangle	^4^
SVR probability	0.84	0.84, 1952	Binomial	Cohort
Mean costs (annual)				
Diagnosis per case identified	$122.95	‐	‐	Cohort
Pre‐treatment for patients not treated, per diagnosed patient	$54.11	$0.89	Normal	Cohort
Full cost of treatment	$716.86	$6.78	Normal	Cohort
Disability weights				
Mild – F0	0	‐‐	‐‐	^38^
Mild – F1	0.25*F4 value	‐‐	‐‐	
Moderate – F2	0.5*F4 value	‐‐	‐‐	
Moderate – F3	0.75*F4 value	‐‐	‐‐	
Compensated cirrhosis – F4	0.114	0.078, 0.159	Uniform	^37^
Decompensated cirrhosis – DCC	0.194	0.123, 0.250	Uniform	^37^
Hepatocellular carcinoma – HCC	0.451	0.307, 0.600	Uniform	^37^
Other parameters				
Cohort initial age	41.3	0.18	Normal	Cohort
Discount rate	0.03	0.00‐0.07	Triangle	

Note

Distribution parameters for PSA (probabilistic sensitivity analysis): Normal: standard deviation, or standard error of the mean when calculated from the study cohort; Triangle: lower, peak, upper; Beta: α [shape1], β [shape2]; Binomial: proportion, sample size; Lognormal: mean and sd on log scale; Uniform: lower, upper.

^#^ Adjusted to reflect the higher proportion of genotype 3 in Pakistan, see Appendix S1.

#### 2.4.3 Treatment costs

HCV screening and treatment costs were estimated directly from programme data using a retrospective, cohort‐based, micro‐costing approach capturing all the provider costs. A detailed review of the treatment protocol and interviews with key staff identified all activities performed and resources utilized in the programme (see Appendix S1).

Information on the types and quantities of resources used for each activity was collected from screening to 12 weeks following treatment completion. The costs borne by the programme included staff time (doctors, nurses, counsellors, laboratory technicians), clinic visits (eligibility assessment, baseline assessments, treatment initiation, on‐treatment monitoring, and SVR12 assessment), diagnostic and laboratory tests, medicines and overheads (buildings, support staff, utilities and consumables).

Primary data were collected on the type and quantity of resources consumed for each activity in the pathway of care for patients. Data on the time spent by staff in providing services were measured using staff time sheets (for patient support nurses and HCV doctors), by interview, or according to total number of patients seen during the costing period. Data on the type and quantity of resources utilized for each patient were collected retrospectively from the Research Electronic Data Capture (RedCap) clinical database.^35^

The 2016 US$ unit prices for resources were applied to estimate the unit cost per activity. These were then applied to patient‐level resource use data to estimate the operational costs for treating each patient. The price for sofosbuvir and daclatasvir reduced due to price negotiations and access campaigns by MSF,^36^ with this negotiated cost (US$282 per 12‐week treatment course of sofosbuvir 400mg plus daclatasvir 60mg) being assumed for the analysis. Key assumptions and unit costs are provided in Supplementary Tables S6, S7 and S8.

Costs of screening included the test for HCV antibodies (anti‐HCV), and when positive, the HCV‐RNA test to confirm chronic infection. The costs also included overheads and staff time for phlebotomy, doing the tests and counselling. The average cost per diagnosis was calculated for the observed anti‐HCV and chronic prevalence at the clinic including costs for patients who received an anti‐HCV and/or HCV‐RNA test but were not reactive.

Information on the healthcare costs for different HCV disease states was not available for Pakistan, and so was not included in the base‐case analysis.

#### 2.4.4 Treatment impact

The impact of the screening and treatment programme was estimated in terms of disability‐adjusted life years (DALYs) averted and numbers of incident liver disease complications prevented (cirrhosis, DC, HCC and liver‐related deaths) compared to no treatment. To estimate DALYs, the 2013 Global Burden of Disease (GBD) disability weights were applied to the HCV disease states in the model^37^ as in previous studies.^38^ We assumed that patients with METAVIR score F0 had no disability, and that compensated cirrhosis (F4) is equivalent to a moderate abdominopelvic problem, with a linear increase in disability from F0 to F4 modelled for F1‐F3. The GBD estimate for DC was used, and the value for metastatic cancer was assumed for HCC (Table 1).

### 2.5 Cost‐effectiveness analysis

In the base‐case analysis, we modelled the costs and DALYs for HCV‐infected persons with and without DAA‐based treatment. The incremental cost‐effectiveness ratio (ICER) was estimated as the ratio of the difference in costs between the screening and treatment programme and the no programme comparator divided by the difference in DALYs. This represents the incremental costs associated with each DALY averted due to the MSF programme compared to if the programme had not occurred, over a lifetime horizon to capture the long‐term progression of the disease. Estimated ICERs were compared against the per capita Gross Domestic Product (GDP) for Pakistan (US$1,442 in 2016), which is commonly used as a willingness‐to‐pay (WTP) threshold for determining whether an intervention is cost‐effective.^39^

### 2.6 Sensitivity Analysis

To quantify the impact of parameter uncertainty on our model results, a probabilistic sensitivity analysis was conducted on all key parameters by simultaneously sampling each parameter 1,000 times from their predefined probability distributions (Table 1) and running the model with each sampled parameter set.

We also examined the effect of changing specific model assumptions through scenario analyses (Supplementary Table S9). These included the following: increasing treatment coverage to 80% (from 51%) of diagnosed patients across all fibrosis stages; assuming reinfection after treatment based on model estimates for Pakistan (3.7 per 1000 person years; none in base‐case) ^4^; SVR of 94% (cure rate among those tested for SVR) instead of 84% in base‐case; reduced cost of sofosbuvir‐daclatasvir ($75 instead of $282 for 12 weeks) to match MSF’s negotiated price for 2019; different age of the cohort (25 or 65 years instead of 42) to evaluate the impact of the background death rate; lower and higher HCV seroprevalence (5.5% reflecting national prevalence or 80%, instead of 33%); shorter time horizon (20 years instead of lifetime); discount rate (0 or 7%, instead of 3%).

Cost savings due to averting onward healthcare costs for HCV‐related disease were not included in the base‐case to present a conservative (more expensive) estimate of the cost‐effectiveness of the intervention. Pakistan has low coverage of health care services, particularly for the poorest members of society,^40^ such as the population targeted by MSF’s clinic in Machar colony. However, additional investment in healthcare towards meeting the sustainable development goals, and related to the drive for HCV elimination is likely to change this in the future, so we incorporated an estimate of averted healthcare costs in the sensitivity analysis. We used healthcare resource use data (self‐reported hospitalizations and outpatient visits) from a survey of HCV‐infected patients diagnosed as part of an MSF treatment intervention in Cambodia adapted for Pakistan, as data on this were not available from the Karachi intervention. Cambodia has similar per capita GDP to Pakistan ($1,270 in 2016) and we applied WHO‐CHOICE health service delivery costs for Pakistan^41^ to the resource use estimates from Cambodia to estimate the healthcare costs of HCV‐related fibrosis and cirrhosis for stages F0‐F4. For DC and HCC, we adjusted costs derived by a WHO taskforce in Cambodia using purchasing power parity adjustment factors (see Appendix S1). We also estimated the impact of accounting for healthcare costs combined with the reduced DAA cost of $75 paid by MSF as of 2019.

Due to the lack of specific disability weights for most HCV disease states, we also considered the effect of using quality‐adjusted life years (QALY) estimates in our ICER calculations. QALY weights came from the same intervention in Cambodia, which surveyed HCV patients using EQ‐5D‐5L to estimate QALY weights by liver disease state.

## 3 RESULTS

### 3.1 Cost of treatment

The average cost of treatment for the 2453 patients that initiated treatment with DAA‐based regimens was $717 (SD $336), with an additional $123 to diagnose each of 4764 patients, and an average of $112 (SD $43) for pre‐treatment assessment for 2,311 patients not treated (Table 2). The major cost driver was the cost of DAAs, contributing 52% of the total cost per initiated treatment from screening through to SVR12 (Table S10), followed by the costs of clinic visits (19%), diagnosis (15%) and laboratory investigations (14%).

**Table 2:** Component costs of HCV treatment intervention. Costs are mean (SD) in 2016 USD

	Clinic costs	Laboratory costs	Other costs	Total per patient
Antibody test	$3.37	$5.65	OraQuick test kit: $8.02	$17.03
Viral load negative result	$8.04	$10.73	GeneXpert consumables (inc. cartridge): $20.90	$39.66
Viral load positive result	$14.28	$10.73	GeneXpert consumables (inc. cartridge): $20.90	$45.90
Total cost per chronic case identified assuming 33% seroprevalence and 78% chronic, n = 4764				$122.95
Cost for pre‐treatment assessment among patients not starting treatment (n = 2311)	$78.56 ($8.55)	$32.97 ($35.13)	‐	$111.53 ($42.64)
Cost of all assessments and treatment among all patients initiated treatment (n = 2453)	$159.87 ($34.33)	$120.75 ($83.24)	DAA drugs: $436.24 ($251.45)	$716.86 ($335.86)

### 3.2 Impact of treatment

**Figure 3:**
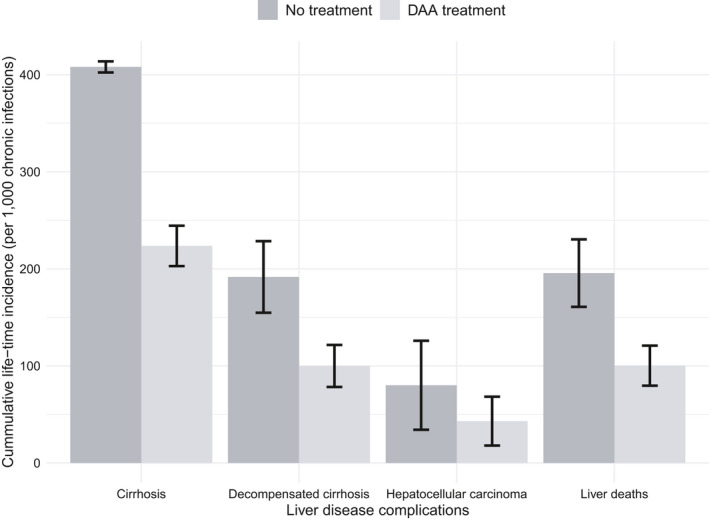
The lifetime incidence of liver‐related complications following DAA‐based treatment compared to no treatment in the intervention cohort. DAA—direct‐acting antivirals. DC, decompensated cirrhosis; HCC, hepatocellular carcinoma. Error bars show one standard deviation

The model estimated that the per patient lifetime cumulative probability of compensated cirrhosis (CC), DC, HCC and liver‐related death without treatment was 42.8%, 32.0%, 32.4% and 33.1%, respectively, decreasing to 27.3%, 17.4%, 17.1% and 17.1% with treatment. This results in the MSF programme preventing 184 cases of CC, 92 cases of DC, 37 cases of HCC and 95 liver‐related deaths, per 1000 patients diagnosed with chronic infection by the programme (Figure 3). Overall, the treatment programme prevents 1.04 DALYs per diagnosed patient.

### 3.3 Base‐case cost‐effectiveness

Under the base‐case projections, the ICER of the treatment programme was $450 per DALY averted (Table 3), much lower than the GDP per capita for Pakistan of $1,442. This is robust to the probabilistic sensitivity analysis, with the ICER in all of the 1,000 simulations falling below US$1,442. The cost‐effectiveness plane and cost‐effectiveness acceptability curve are shown in Supplementary Materials Figure S2 and S3.

**Table 3:** Base‐case costs per person with chronic HCV infection, effects and incremental cost‐effectiveness ratio (ICER) for DAA‐based treatment compared to no treatment

	Costs, US$		Health outcomes		ICER
Treatment strategy	Total cost	Incremental costs	DALYs	DALYs averted	US$/DALY averted
No treatment	0	–	2.26	–	–
DAA‐based treatment	$466	$466	1.23	1.04	$450

### 3.4 Deterministic sensitivity analysis

**Figure 4:**
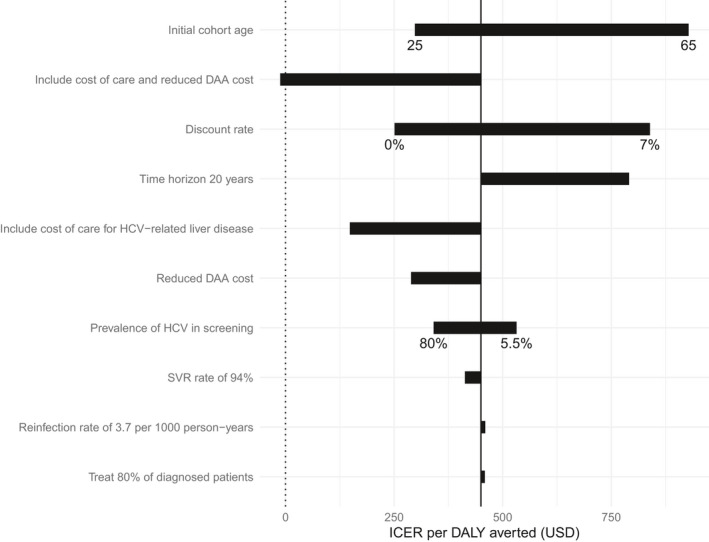
Tornado diagram for univariate scenario analyses showing the impact of changes in parameter values on the base case incremental cost‐effectiveness ratio (ICER = $450/DALY, represented by the solid vertical line)

DAA‐based treatment remained cost‐effective (ICER<$1442/DALY averted) under all evaluated scenarios in the univariate analysis (Figure 4). Assuming a treatment coverage of 80% of all diagnosed patients resulted in higher incremental costs and health benefits, with a slight increase in the ICER ($459/DALY averted). A lower seroprevalence of HCV (5.5%) increased the cost, but the programme remained cost‐effective at an ICER of $532/DALY averted. Modelling an older cohort (65 years) increased the ICER to $928/DALY averted. Including healthcare costs due to HCV made the intervention cost‐effective at the opportunity cost threshold ($148/DALY averted), and cost‐saving (‐$12/DALY) when combined with the reduced DAA cost ($75 per 12‐week treatment). The programme remained cost‐effective when using QALYs as health outcomes ($538/QALY gained). Other sensitivity analyses (Figure 4) on DAA costs, discount rate, time horizon and reinfection rate suggested the cost‐effectiveness projections were robust to these parameter changes (ICER<$1000/DALY averted).

## 4 DISCUSSION

This study demonstrates that the MSF testing and DAA‐based treatment programme in a primary healthcare setting in Karachi, Pakistan are potentially cost‐effective for reducing the HCV disease burden in this setting when compared to no treatment, and with reduced DAA costs, may be cost‐saving when averted healthcare costs are accounted for.

Although good treatment outcomes have been demonstrated for this^33^ and other treatment programmes in LMICs,^42^ there are concerns about the costs and sustainability of DAA‐based treatment programmes in these settings.^43^, ^44^, ^45^ Therefore, cost‐effectiveness analyses are important for informing policy makers on the value of these programmes in resource‐limited settings. Unfortunately, most evidence on the cost‐effectiveness of HCV treatment comes from upper‐middle and high‐income countries,^46^, ^47^, ^48^ and so our analysis is important in providing evidence that DAA‐based testing and treatment programmes could represent good value for money when integrated in primary healthcare settings in LMICs.^19^

This study draws major strength from the use of ‘real‐world’ data on patients screened, diagnosed, and treated as part of an on‐going programme in Pakistan. This enabled collection of patient‐level data on resource use to estimate the full costs associated with DAA‐based testing and treatment of HCV. We also used outcome (testing and linkage data) and effectiveness data (SVR12 rates) from the programme, so representing the ‘real‐world implementation’ of DAA‐based regimens. This contrasts with previous cost‐effectiveness studies in LMICs, where cost estimates have been largely based on expert opinion^19^ and treatment efficacy data have come from clinical trials.^19^, ^49^ This could be far from representative of ‘real‐world’ costs and outcomes, especially relating to the non‐patient‐related costs of the intervention and the level of linkage between testing and treatment.

Despite these strengths, several potential limitations exist in our evaluation. Firstly, the use of a static Markov model only characterizes the direct patient‐level benefits of the programme and fails to capture prevention benefits,^50^, ^51^, ^52^ potentially leading to underestimation of cost‐effectiveness. This choice was made due to the low assumed risk of reinfection in generalized epidemic settings,^4^ with sensitivity analyses confirming that its inclusion does not affect our results. Secondly, we used costs and outcome data from a single programme implemented by an international organization in a high prevalence setting, potentially limiting the generalizability of our results. It is possible that our costs may be higher than government run programmes, which can have simpler pathways of care, and our outcomes may also be different because of this. Our results are likely to be most relevant to other high prevalence settings in South Asia, although sensitivity analyses suggest the intervention will still be cost‐effective at lower HCV screening prevalences. Thirdly, we did not estimate the healthcare costs of HCV‐related disease in Pakistan, and this omission in the base‐case analysis will have resulted in an underestimation of the cost savings resulting from the programme. When the costs of HCV‐related care were included based on resource use data from Cambodia, the cost‐effectiveness of the programme improved dramatically (ICER = US$148/DALY averted). Data on the costs of care in this setting will enable a more precise estimation of the cost‐effectiveness of such programmes. Fourthly, most disability weights used are not specific to HCV. Reassuringly, when we performed a sensitivity analysis using QALY weights derived from and HCV treatment programme in Cambodia, the result was similarly cost‐effective (ICER=$US538/QALY gained). Lastly, we compared the intervention to undertaking no testing and treatment which may not represent the evolving availability of HCV services in this setting. Unfortunately, there were no data on the costs and outcomes of other treatment options and so we could not consider a different comparator.

In this study, we compared the estimated ICER to a WTP threshold based on GDP per capita, as is widely used in the literature, despite WHO and others stating the limitations of using these thresholds for national health decisions.^53^, ^54^ A recently proposed estimate of thresholds based on health opportunity cost suggests that $133 ‐ $175/DALY would be a more appropriate threshold for Pakistan.^55^ With this threshold, our baseline estimate would no longer be considered cost‐effective, but inclusion of cost of care brings the ICER to within range ($148/DALY).

To date, a small number of studies evaluating the cost‐effectiveness of DAA‐based regimens in LMICs have reported promising findings.^19^, ^20^, ^23^ In India, an analysis found DAA‐based HCV testing and treatment to be cost‐saving,^19^ but India‐derived costs or treatment outcome data were not used. In addition, savings in health care costs, derived from the USA, may have been too high for low‐income settings. In Egypt, screening and treatment were found to be cost‐effective but the intervention used a combination of DAA and interferon‐based treatment which are no longer widely used.^20^, ^49^ Our study provides important new data from a ‘real‐world’ economic evaluation of HCV testing and treatment in a resource‐limited setting—providing the first real evidence that testing and DAA‐based treatment can be highly cost‐effective in LMICs. This is the first study to evaluate a primary health clinic‐based intervention in LMIC.

The favourable cost‐effectiveness of the HCV testing and treatment programme has important implications for Pakistan. It provides important data that supports the scale‐up of testing and treatment, which recent modelling suggests is urgently needed for containing the current increasing HCV epidemic.^4^, ^56^ The generalized nature of the HCV epidemic in Pakistan requires decentralization of services in order to reach all the affected populations, particularly among marginalized groups that may not have regular access to healthcare facilities, such as individuals living in informal settlement areas. Our results support the countrywide expansion of decentralized screening and treatment strategies among these populations across Pakistan, with our cost estimates being useful for determining the likely costs of such a strategy.^56^ Further simplification of the pathway of care, reductions in cost of screening, diagnosis and DAA prices will further improve access and cost‐effectiveness.

## CONFLICT OF INTEREST

PV has received unrestricted research grants from Gilead unrelated to this study and honoraria from Gilead and Abbvie. JGW has received a research grant from Gilead unrelated to this study. GGK, KA, YW, HZ, RVB, GF, RA, DD, CF and AL were employed by MSF and/or by Epicentre, an association created by MSF in 1986 to provide epidemiological expertise to underpin MSF operations. They participated in planning the study, carrying out the research and writing the report. The other authors declared no conflicts of interest.

## Supporting information

Supplementary Material
