# Supplementary material for: Cost‐effectiveness of screening and treatment using direct‐acting antivirals for chronic Hepatitis C virus in a primary care setting in Karachi, Pakistan
Source: J Viral Hepat. 2020 Nov 4;28(2):268–78. doi: 10.1111/jvh.13422 (PMC7821258; doi:10.1111/jvh.13422)
Supplement: Supplementary file 1 — Supplementary Material [file JVH-28-268-s001.docx]

Supplementary Information

Cost-effectiveness of screening and treatment using direct-acting antivirals for chronic Hepatitis C virus in a primary care setting in Karachi, Pakistan

Nyashadzaishe Mafirakureva^1^, Aaron G Lim^1^, Gul Ghuttai Khalid^3^, Khawar Aslam^3^, Linda Campbell^1^, Hassaan Zahid^3^, Rafael Van den Bergh^2^, Gregoire Falq^4^, Camille Fortas^4^, Yves Wailly^2^, Rosa Auat^2^, Dmytro Donchuk^2^, Anne Loarec^4^, Joanna Coast^1^, Peter Vickerman ^1,5^, Josephine G. Walker^1^

1. Population Health Sciences, Bristol Medical School, University of Bristol
2. Operational Center Brussels, Médecins Sans Frontières, Brussels, Belgium
3. Operational Center Brussels, Médecins Sans Frontières, Islamabad, Pakistan
4. Epicentre, Paris, France
5. NIHR Health Protection Research Unit in Behavioural Science and Evaluation at University of Bristol

Table of Contents

[Appendix S1: Supplementary Methods 4](#_Toc54777648)

[Cost analysis of HCV treatment intervention 4](#_Toc54777649)

[Design 4](#_Toc54777650)

[Identifying resources used 4](#_Toc54777651)

[Measuring resource utilization 4](#_Toc54777652)

[Valuation of resources 5](#_Toc54777653)

[HCV visit costs 5](#_Toc54777654)

[HCV treatment-related laboratory costs 5](#_Toc54777655)

[DAA medicine costs 6](#_Toc54777656)

[HCV diagnosis costs 6](#_Toc54777657)

[Estimation of health care costs for hepatitis C related disease states 6](#_Toc54777658)

[Adjustment of HCV disease progression 7](#_Toc54777659)

[Consolidated Health Economic Evaluation Reporting Standards (CHEERS) checklist 8](#_Toc54777660)

[Supplementary Tables 11](#_Toc54777661)

[Table S1. Distribution of cohort of diagnosed patients by fibrosis stages (n=4,541). 11](#_Toc54777662)

[Table S2. Baseline characteristics of patients in the cohort 11](#_Toc54777663)

[Table S3. Characteristics of patients who completed treatment 13](#_Toc54777664)

[Table S4. Unit Costs for Patient Services for Pakistan[3] 14](#_Toc54777665)

[Table S5. Correlation of Hepatitis C Virus Disease Stage by Invasive (Liver Biopsy Staging) and Non-invasive (APRI Scores). Adapted from [10] 14](#_Toc54777666)

[Table S6. Staff types and staff times per patient for the different activities in the HCV screening and treatment intervention. 15](#_Toc54777667)

[Table S7. Activities, resources and estimated unit costs 16](#_Toc54777668)

[Table S8. Unit costs for visits, laboratory tests, test kits and medicines 18](#_Toc54777669)

[Table S9. Variables used in the univariate sensitivity analysis 19](#_Toc54777670)

[Table S10. Proportion of total costs per patient diagnosed, treated, or cured attributable to components of treatment costs, Mean (SD) 19](#_Toc54777671)

[Supplementary Figures 20](#_Toc54777672)

[Figure S1: Diagnostic and treatment algorithm for patients in Hepatitis C treatment program. 21](#_Toc54777673)

[Figure S2: Cost-effectiveness plane 22](#_Toc54777674)

[Figure S3: Cost-effectiveness acceptability curve 23](#_Toc54777675)

[References 24](#_Toc54777676)

# Appendix S1: Supplementary Methods

## Cost analysis of HCV treatment intervention

### Design

The costs of HCV screening and treatment were estimated directly from MSF program data using a retrospective, cohort-based, micro-costing approach from the provider’s perspective. Data on resource use and costs were retrospectively obtained from MSF’s financial records over a 17-month observation period (January 2016 – May 2017), and results are presented in 2016 US dollars. This represents a time period in which the intervention had become fairly stable (first cohort of patients initiated in 2015). The cost analyses followed the standard techniques for conducting micro-cost analyses, which involve the identification, quantification and valuation of all the resources (direct medical and non-medical) used in the screening, diagnosis, treatment and follow-up for each patient in the cohort. We gathered both financial and economic costs, however only economic costs are reported to capture the likely opportunity costs associated with the intervention. The total costs of the intervention included intervention set-up and implementation costs. Research-related costs were identified and excluded from the analysis. Using an ingredients approach, unit costs were applied to patient-level resource use (in terms of type and frequency of visit) to obtain the total cost for each patient.

### Identifying resources used

A detailed review of the treatment protocol and interviews with key technical staff involved in the planning, implementation and coordination of the intervention were performed to identify all the activities and resources utilized in the treatment of patients with chronic HCV in the Machar colony in Karachi, Pakistan. The main activities in the intervention included HCV antibody testing using a rapid diagnostic test, confirmatory testing for HCV chronic infection using an RNA based test, patient counselling/education, baseline medical assessment for treatment eligibility, baseline laboratory work up, DAA treatment initiation, treatment follow-up, treatment monitoring laboratory tests, routine management of medical problems, on-treatment referrals to other medical services, post-treatment follow up and SVR assessment. The resources identified included staff time (nurse counsellor, medical doctor, laboratory technician), materials (test kits, consumables), laboratory tests (HCV antibody, HCV RNA, HCV genotype), DAA medicines and overheads. Overheads included facility/buildings, utilities, support staff, coordination staff, vehicles, training, travel, medical and laboratory supplies, non-medical supplies, freight and clearance.

### Measuring resource utilization

Primary data was collected on the exact number and type of resources consumed in the treatment program. Detailed patient-level data, including the number and type of visits, clinical examinations, laboratory investigations, treatment regimens, and treatment outcomes were extracted from data collected during the study using a Research Electronic Data Capture (REDCap)[1] database (January 2015 – October 2018). The amount of time spent by staff providing services in the program was estimated for each activity using staff time sheets and interviews with the relevant staff. Overhead and administrative costs associated with the HCV treatment program were allocated using the step-down costing approach. For example, staff numbers in each department were used to allocate management and administrative costs, service statistics were used to allocate shared recurrent resources, interviews with key personnel (mostly managers or supervisors) were performed to determine ratios for allocating coordination costs and room space was used to allocate buildings space, utilities and building maintenance.

### Valuation of resources

Valuation of the resources used in the treatment program was based on MSF program financial records and information provided by program staff (finance, logistics, and pharmacy staff). The most up-to-date unit prices/costs were applied to patient-level data on resource use to estimate the costs of treating each patient. Unit costs for supplies and consumables, including test kits, were obtained from MSF financial records and supplemented with interviews with key personnel (finance, logistics, and program managers). Unit costs for valuing staff time were estimated based on staff salaries information provided by MSF. Valuation of the DAAs was based on the prices paid for the medicines by MSF at the time of purchase. Unit costs for the outsourced laboratory tests were obtained from the external laboratory through MSF. When unavailable, the unit cost for an activity was estimated using micro-costing. Building costs (rentals) and floor layout plans were provided by the MSF’s logistics department. Information on the acquisition costs and replacement values for capital items were gathered from the project’s program records. Equivalent annual costs for capital items (equipment and furniture) were estimated based on the expected service lives using a discount rate of 3%.

All historical costs were adjusted for inflation to 2016 prices using the Consumer Price Indices obtained for Pakistan. Unit prices were gathered in both the local currency (Pakistani rupee) and USD. Local currency prices were converted to the USD currency using the average market-based exchange rate (1 USD = 133 Pakistani rupee). The per unit overhead costs for each activity were estimated by dividing the annual total cost for the activity by the annual total number of units of output (for example, number of patient visits or patients). The cost of each activity is the sum of the costs for all the resources used in executing that activity, i.e, labour, consumables and overheads. The activity costs were multiplied by the number of times a patient received each activity and summed to give an estimate of the total cost per patient. The total costs comprised of the following categories: HCV diagnosis costs, HCV-related visit costs, HCV treatment related laboratory costs and DAA costs.

#### HCV visit costs

HCV-related visits comprised of all visits made by patients in preparation for, during and after treatment. These included baseline assessments, treatment initiation, on-treatment follow up, end of treatment, post-treatment follow-up and SVR assessment visits. Each visit cost included the cost of staff time specific to the visit (estimated using staff time sheets and interviews) and space/materials depending on which area of the clinic was utilized (laboratory visit, medical visit, counselling visit, pharmacy visit). For each of these locations, the visit cost incorporated recurrent costs (support personnel costs, medicines (excluding HCV), medical and laboratory supplies, non-medical supplies, transport operating costs, building rental and insurance, maintenance, utilities and bills, freight and clearance, travel, and training) and capital costs (buildings, vehicles, medical equipment, laboratory equipment, cold chain equipment, non-medical equipment, construction and rehabilitation, and furniture). Building space for each location was determined through site maps, visual inspection and interviews with the logistics manager and allocated as HCV-related by proportion of HCV consultations. Support personnel costs for each category (coordination, administration, human resources, support staff), were determined by their level of involvement in HCV-related activities and allocated using proportion of staff, budget, floor space, or consultations.

#### HCV treatment-related laboratory costs

Laboratory costs included all laboratory tests and investigations performed for each patient in preparation for, during and after treatment according to the MSF treatment protocol and were obtained from MSF financial records. The cost for the GeneXpert PCR system was estimated using a micro-costing and included the cost of the equipment cost, reagents cost, installation and maintenance and staff time. The costs of laboratory tests contracted outside of the program were gathered from hospital price lists and from invoices billed to MSF.

#### DAA medicine costs

Unit costs for DAAs were determined from detailed MSF invoices and interview with the Hepatitis C pharmacist at MSF’s Access Campaign. DAA costs for each patient were calculated based on the patients-specific treatment regimen and the length of treatment obtained from the REDCap database.

#### HCV diagnosis costs

These include the costs of screening for HCV antibodies, and when positive, HCV-RNA screening test to confirm chronic infection. The average cost per diagnosis was calculated for the observed HCV antibody and chronic prevalence at the clinic including costs for patients who received an HCV antibody and/or HCV-RNA test but were not reactive. This represented the full cost of HCV case-finding.

To estimate the costs of diagnosis, we estimated the costs of the screening test for HCV antibodies, and when positive, an HCV-RNA screening test to confirm chronic infection. All these costs include staff time (for phlebotomy, doing the tests and counselling) and overhead costs. These were summed up to get the total unit costs for a negative or positive RDT and RNA test. The average cost per diagnosis was then calculated for the observed HCV antibody (33%) and chronic prevalence (73.8%) at the clinic including costs for patients who received an HCV antibody and/or HCV-RNA test but were not reactive. The following formulae were applied to arrive at the full cost of HCV case-finding in this cohort.

- Cost of RDT test = cost of OPD consultation + cost of HCV RDT kit
- Cost of PCR negative = cost of PCR result negative counselling visit + cost of GeneXpert test
- Cost of PCR positive = cost of PCR result positive counselling visit + cost of GeneXpert test
- Cost per diagnosis = [Cost of RDT positive*observed HCV antibody prevalence + Cost of RDT negative*(1- observed HCV antibody prevalence) + observed HCV antibody prevalence* Cost of PCR positive* observed chronic HCV prevalence + observed HCV antibody prevalence* PCR negative*(1- observed chronic HCV prevalence)] / (observed HCV antibody prevalence*observed chronic HCV prevalence)

## Estimation of health care costs for hepatitis C related disease states

Due to non-availability of healthcare resource use data specific for Pakistan, we used resource use data (visits to the health centre, hospital outpatient visits or hospitalizations) from a HCV cross-sectional patient survey undertaken in Cambodia[2] and applied WHO-CHOICE Health service delivery costs for Pakistan[3] to estimate the healthcare costs of HCV for stages F0-F4. The survey involved the use of a questionnaire to ask patients to recall health care resource use in the past 6 months prior to enrolment into the MSF HCV clinic. These included visits to primary, secondary or tertiary health care facilities. Estimates of unit costs for patient services for Pakistan were applied to resource use data to obtain the costs of care for HCV Metavir stages F0 – F4. These costs were adjusted for inflation by applying the relevant consumer price indices for 2005 and 2016. Resource use information was not available for decompensated cirrhosis and hepatocellular carcinoma, and we applied costs derived by a WHO taskforce in Cambodia by adjusting them to Pakistan levels using the purchasing power parity adjustment factors.

## Adjustment of HCV disease progression

Transition probabilities for each disease state were sourced from literature [4-6] (Table 2). These transition probabilities were adjusted to reflect the higher proportion (80%) of genotype 3 in the cohort (and Pakistan in general[7]), which has faster disease progression[8, 9]. Data from a large cohort in the USA showed that HCV genotype 3 is associated with an increased relative risk of 1.31 [1.22–1.39] for disease progression to compensated or decompensated cirrhosis, and a relative risk of 1.80 [1.61–2.03] for the development of HCC [8]. Applying this to 80% of infected individuals gives risk ratios of 1.17 (95% 1.115-1.195) for progression to cirrhosis and 1.41 (95% CI 1.287-1.45) for progression to HCC [9].

# Consolidated Health Economic Evaluation Reporting Standards (CHEERS) checklist

| Section/item | Item No | Recommendation | Reported on page No/ line No |
| --- | --- | --- | --- |
| Title and abstract |  |  |  |
| Title | 1 | Identify the study as an economic evaluation or use more specific terms such as “cost-effectiveness analysis”, and describe the interventions compared. | Page 1 |
| Abstract | 2 | Provide a structured summary of objectives, perspective, setting, methods (including study design and inputs), results (including base case and uncertainty analyses), and conclusions. | Page 2 |
| Introduction |  |  |  |
| Background and objectives | 3 | Provide an explicit statement of the broader context for the study.  Present the study question and its relevance for health policy or practice decisions. | Page 3 |
| Methods |  |  |  |
| Target population and subgroups | 4 | Describe characteristics of the base case population and subgroups analysed, including why they were chosen. | Page 4  Figure 1  Supplementary Table S2, S3 |
| Setting and location | 5 | State relevant aspects of the system(s) in which the decision(s) need(s) to be made. | Page 4 |
| Study perspective | 6 | Describe the perspective of the study and relate this to the costs being evaluated. | Page 3 |
| Comparators | 7 | Describe the interventions or strategies being compared and state why they were chosen. | Page 3 |
| Time horizon | 8 | State the time horizon(s) over which costs and consequences are being evaluated and say why appropriate. | Page 6 |
| Discount rate | 9 | Report the choice of discount rate(s) used for costs and outcomes and say why appropriate. | Page 4 |
| Choice of health outcomes | 10 | Describe what outcomes were used as the measure(s) of benefit in the evaluation and their relevance for the type of analysis performed. | Page 6 |
| Measurement of effectiveness | 11a | Single study-based estimates: Describe fully the design features of the single effectiveness study and why the single study was a sufficient source of clinical effectiveness data. | Page 4-5 |
|  | 11b | Synthesis-based estimates: Describe fully the methods used for identification of included studies and synthesis of clinical effectiveness data. | Not applicable |
| Measurement and valuation of preference based outcomes | 12 | If applicable, describe the population and methods used to elicit preferences for outcomes. | Not applicable |
| Estimating resources and costs | 13a | Single study-based economic evaluation: Describe approaches used to estimate resource use associated with the alternative interventions. Describe primary or secondary research methods for valuing each resource item in terms of its unit cost. Describe any adjustments made to approximate to opportunity costs. | Not applicable |
|  | 13b | Model-based economic evaluation: Describe approaches and data sources used to estimate resource use associated with model health states. Describe primary or secondary research methods for valuing each resource item in terms of its unit cost. Describe any adjustments made to approximate to opportunity costs. | Page 4  Page 5-6  Supplementary materials Tables S6-S8 |
| Currency, price date, and conversion | 14 | Report the dates of the estimated resource quantities and unit costs. Describe methods for adjusting estimated unit costs to the year of reported costs if necessary. Describe methods for converting costs into a common currency base and the exchange rate. | Page 6  Supplementary materials |
| Choice of model | 15 | Describe and give reasons for the specific type of decision-analytical model used. Providing a figure to show model structure is strongly recommended. | Page 4  Figure 1 |
| Assumptions | 16 | Describe all structural or other assumptions underpinning the decision-analytical model. | Page 5 |
| Analytical methods | 17 | Describe all analytical methods supporting the evaluation. This could include methods for dealing with skewed, missing, or censored data; extrapolation methods; methods for pooling data; approaches to validate or make adjustments (such as half cycle corrections) to a model; and methods for handling population heterogeneity and uncertainty. | Not applicable |
| Results |  |  |  |
| Study parameters | 18 | Report the values, ranges, references, and, if used, probability distributions for all parameters. Report reasons or sources for distributions used to represent uncertainty where appropriate. Providing a table to show the input values is strongly recommended. | Table 1 |
| Incremental costs and outcomes | 19 | For each intervention, report mean values for the main categories of estimated costs and outcomes of interest, as well as mean differences between the comparator groups. If applicable, report incremental cost-effectiveness ratios. | Page 7-8  Tables 2&3 |
| Characterising uncertainty | 20a | Single study-based economic evaluation: Describe the effects of sampling uncertainty for the estimated incremental cost and incremental effectiveness parameters, together with the impact of methodological assumptions (such as discount rate, study perspective). | Not applicable |
|  | 20b | Model-based economic evaluation: Describe the effects on the results of uncertainty for all input parameters, and uncertainty related to the structure of the model and assumptions. | Page 8  Figure 4 |
| Characterising heterogeneity | 21 | If applicable, report differences in costs, outcomes, or cost-effectiveness that can be explained by variations between subgroups of patients with different baseline characteristics or other observed variability in effects that are not reducible by more information. | Page 7 |
| Discussion |  |  |  |
| Study findings, limitations, generalisability, and current knowledge | 22 | Summarise key study findings and describe how they support the conclusions reached. Discuss limitations and the generalisability of the findings and how the findings fit with current knowledge. | Page 8  Page 9 |
| Other |  |  |  |
| Source of funding | 23 | Describe how the study was funded and the role of the funder in the identification, design, conduct, and reporting of the analysis. Describe other non-monetary sources of support. | Page 1 |
| Conflicts of interest | 24 | Describe any potential for conflict of interest of study contributors in accordance with journal policy. In the absence of a journal policy, we recommend authors comply with International Committee of Medical Journal Editors recommendations. | Page 1 |

# Supplementary Tables

## Table S1. Distribution of cohort of diagnosed patients by fibrosis stages (n=4,541).

F0-F4 are METAVIR scores estimated using APRI scores. Patients with unknown fibrosis stage were not included in the Markov model cohort (223, of whom 4 were treated).

| Disease stage | N | % of total | Treated | % of group treated |
| --- | --- | --- | --- | --- |
| F0 | 1112 | 24.5 | 127 | 11.4 |
| F1 | 580 | 12.8 | 200 | 34.5 |
| F2 | 1514 | 33.3 | 1117 | 73.8 |
| F3 | 824 | 18.1 | 639 | 77.5 |
| F4 | 511 | 11.3 | 366 | 71.6 |

## Table S2. Baseline characteristics of patients in the cohort

| Variable | Not started treatment (N=2311) | Started treatment (N=2453) | Total (N=4764) |
| --- | --- | --- | --- |
| METAVIR fibrosis stage | | | |
| F0 | 985 (42.6%) | 127 (5.2%) | 1112 (23.3%) |
| F1 | 380 (16.4%) | 200 (8.2%) | 580 (12.2%) |
| F2 | 397 (17.2%) | 1117 (45.5%) | 1514 (31.8%) |
| F3 | 185 (8.0%) | 639 (26.0%) | 824 (17.3%) |
| F4 | 145 (6.3%) | 366 (14.9%) | 511 (10.7%) |
| Missing | 219 (9.5%) | 4 (0.2%) | 223 (4.7%) |
| Age (years) | | | |
| Mean (SD) | 39.9 (12.5) | 42.6 (12.1) | 41.3 (12.4) |
| Range | 18 - 96 | 18 - 84 | 18 - 96 |
| Gender | | | |
| Male | 845 (36.6%) | 1016 (41.4%) | 1861 (39.1%) |
| Female | 1464 (63.4%) | 1437 (58.6%) | 2901 (60.9%) |
| Missing | 2 | 0 | 2 |
| Genotype | | | |
| 1 | 12 (0.5%) | 19 (0.8%) | 31 (0.7%) |
| 2 | 7 (0.3%) | 12 (0.5%) | 19 (0.4%) |
| 3 | 56 (2.4%) | 207 (8.4%) | 263 (5.5%) |
| 4 | 0 (0.0%) | 1 (0.0%) | 1 (0.0%) |
| Missing | 2236 (96.8%) | 2214 (90.3%) | 4450 (93.4%) |

## Table S3. Characteristics of patients who completed treatment

| Variable | Not reached SVR12 (N=332) | Reached SVR12 (N=1709) | Total (N=2041*) |
| --- | --- | --- | --- |
| METAVIR fibrosis stage, n (%) | | | |
| F0 | 24 (7.2%) | 73 (4.3%) | 97 (4.8%) |
| F1 | 27 (8.1%) | 147 (8.6%) | 174 (8.5%) |
| F2 | 155 (46.7%) | 828 (48.4%) | 983 (48.2%) |
| F3 | 75 (22.6%) | 449 (26.3%) | 524 (25.7%) |
| F4 | 50 (15.1%) | 212 (12.4%) | 262 (12.8%) |
| Missing | 1 (0.3%) | 0 (0.0%) | 1 (0.0%) |
| Age (years) | | | |
| Mean (SD) | 42.6 (12.6) | 42.7 (11.6) | 42.7 (11.8) |
| Gender, n (%) | | | |
| Male | 151 (45.5%) | 670 (39.2%) | 821 (40.2%) |
| Female | 181 (54.5%) | 1039 (60.8%) | 1220 (59.8%) |
| Genotype, n (%) | | | |
| 1 | 1 (0.3%) | 17 (1.0%) | 18 (0.9%) |
| 2 | 1 (0.3%) | 10 (0.6%) | 11 (0.5%) |
| 3 | 12 (3.6%) | 179 (10.5%) | 191 (9.4%) |
| 4 | 0 (0.0%) | 1 (0.1%) | 1 (0.0%) |
| Missing | 318 (95.8%) | 1502 (87.9%) | 1820 (89.2%) |
| Previous HCV treatment regimen, n (%) | | | |
| Naive | 277 (90.8%) | 1130 (80.8%) | 1407 (82.6%) |
| DAA | 2 (0.7%) | 3 (0.2%) | 5 (0.3%) |
| INF+RBV | 24 (7.9%) | 204 (14.6%) | 228 (13.4%) |
| PEG | 0 (0.0%) | 14 (1.0%) | 14 (0.8%) |
| RBV | 2 (0.7%) | 47 (3.4%) | 49 (2.9%) |
| Missing | 27 | 311 | 338 |
| First treatment regimen in intervention, n (%) | | | |
| SOF+DAC30 | 1 (0.3%) | 0 (0.0%) | 1 (0.0%) |
| SOF+DAC60 | 297 (89.5%) | 1123 (65.7%) | 1420 (69.6%) |
| SOF+DAC60+RBV1000 | 8 (2.4%) | 103 (6.0%) | 111 (5.4%) |
| SOF+DAC60+RBV1200 | 2 (0.6%) | 11 (0.6%) | 13 (0.6%) |
| SOF+DAC60+RBV600 | 4 (1.2%) | 16 (0.9%) | 20 (1.0%) |
| SOF+DAC60+RBV800 | 6 (1.8%) | 56 (3.3%) | 62 (3.0%) |
| SOF+DAC60+VEL | 1 (0.3%) | 0 (0.0%) | 1 (0.0%) |
| SOF+DAC90 | 0 (0.0%) | 1 (0.1%) | 1 (0.0%) |
| SOF+RBV1000 | 7 (2.1%) | 265 (15.5%) | 272 (13.3%) |
| SOF+RBV1000+PEG | 0 (0.0%) | 2 (0.1%) | 2 (0.1%) |
| SOF+RBV1200 | 0 (0.0%) | 51 (3.0%) | 51 (2.5%) |
| SOF+RBV600 | 1 (0.3%) | 2 (0.1%) | 3 (0.1%) |
| SOF+RBV800 | 5 (1.5%) | 78 (4.6%) | 83 (4.1%) |
| SOF+RBV800+PEG | 0 (0.0%) | 1 (0.1%) | 1 (0.0%) |
| First treatment length (days) | | | |
| Median (Q1, Q3) | 91 (85, 171.0) | 97 (86, 172) | 95 (86, 172) |
| Re-treated | | | |
| FALSE | 331 (99.7%) | 1676 (98.1%) | 2007 (98.3%) |
| TRUE | 1 (0.3%) | 33 (1.9%) | 34 (1.7%) |
| DAA=directly-acting antivirals, INF=interferon, RBV=ribavirin [600,800,1000, or 1200 mg], SOF=sofosbuvir, DAC= daclatasvir [30,60, or 90 mg], VEL=velpatasvir, PEG= pegylated interferon. *Of 2,453 patients who started treatment, 203 patients were still on treatment, 7 died, 13 stopped for medical reasons or transferred out, and 189 were lost to follow up during treatment | | | |

## Table S4. Unit Costs for Patient Services for Pakistan[3]

|  | 2005 $ | 2016 $ |
| --- | --- | --- |
| Cost per bed day by hospital level* | | |
| Primary | 21.24 | 58.20 |
| Secondary | 27.71 | 75.93 |
| Tertiary | 37.85 | 103.71 |
| Cost per outpatient visit by hospital level* | | |
| Primary | 6.09 | 16.69 |
| Secondary | 8.63 | 23.65 |
| Tertiary | 12.77 | 34.99 |
|  |  |  |
| Cost per visit at health centre by population coverage for a 20 minute visit** | | |
| 50% | 7.52 | 20.60 |
| 80% | 7.52 | 20.60 |
| 95% | 8.46 | 23.18 |
| * public facility, 80% occupancy rate, excludes drugs and diagnostics  ** public facility, at different population coverage, excludes drugs and diagnostics  Consumer price index 2005 – 55.317  Consumer price index 2016 – 150.753 | | |

## Table S5. Correlation of Hepatitis C Virus Disease Stage by Invasive (Liver Biopsy Staging) and Non-invasive (APRI Scores). Adapted from [10]

| Degree of Fibrosis (Stage) | Metavir | Mean APRI Score (95% CI) |
| --- | --- | --- |
| No fibrosis (F0) (n = 267) | F0 | 0.35 (0.32–0.38) |
| Fibrous portal expansion (F1) (n = 555) | F1 | 0.50 (0.47–0.53) |
| Few bridges or septa (F2) (n = 648) | F2 | 0.67 (0.63–0.71) |
| Numerous bridges or septa (F3) (n = 394) | F3 | 1.06 (.98–1.15) |
| Cirrhosis (F4) (n = 508) | F4 | 1.77 (1.63–1.92) |
| Abbreviations: APRI, aspartate aminotransferase–to-platelet ratio index; CI, confidence interval; IASL, International Association for the Study of the Liver. | | |

## Table S6. Staff types and staff times per patient for the different activities in the HCV screening and treatment intervention.

| Activity | Personnel | Mean time in minutes (SD) |
| --- | --- | --- |
| **Out-patients department (OPD) visit** | | |
| OPD consultation | OPD Medical doctor | 3*† |
| **Counselling sessions** | | |
| PCR negative results counselling | Patient support nurse | 16 (5.6) |
| PCR positive results counselling | Patient support nurse | 24 (7.7) |
| Treatment eligibility | Patient support nurse | 15 (7.4) |
| Treatment initiation | Patient support nurse | 17 (7.2) |
| Lifestyle session (Treatment follow-up/end of treatment) | Patient support nurse | 13 [n=1] |
| SVR12 | Patient support nurse | 14 (4.4) |
| **HCV clinic visits** | | |
| Baseline initial assessment | Medical doctor | 28 (5.8) |
| Baseline subsequent assessment | Medical doctor | 15 (4.8) |
| Treatment initiation consultation | Medical doctor | 16 (4.8) |
| Treatment follow-up consultation | Medical doctor | 10 (2.8) |
| End of treatment | Medical doctor | 11 [n=1] |
| SVR12 | Medical doctor | 15 (7.7) |
| Family planning consultation | Maternal and child health nurse | 20* |
| **Laboratory visits** | | |
| HCV Rapid diagnostic test | Laboratory technician | 7.5* |
| Phlebotomy | Laboratory technician | 10.0* |
| GeneXpert | Laboratory technician | 69*‡ |
| OPD=out-patient department, RDT=rapid diagnostic test, PCR=polymerase chain reaction, SVR12=sustained virological response, SD=standard deviation. *Based on interview so no SD available (all other estimates from timesheets). †Incremental time added to OPD consultation for HCV screening. | | |

## Table S7. Activities, resources and estimated unit costs

| Clinic visits | Ingredients | Type | Unit cost (US$) | |
| --- | --- | --- | --- | --- |
|  |  |  | Financial | Economic |
| Out-patients department (OPD) consultation | OPD receptionist | Staff time | 0.12 | 0.12 |
|  | OPD nursing | Staff time | 1.05 | 1.05 |
|  | OPD medical Doctor | Staff time | 0.39 | 0.39 |
|  | OPD clinic visit | Space/Materials | 1.81 | 1.81 |
| HCV Rapid Diagnostic Test (RDT) | Laboratory technician | Staff time | 0.40 | 0.40 |
|  | Laboratory visit | Space/Materials | 5.23 | 5.25 |
| GeneXpert HCV viral load test | GeneXpert consumables (including cartridge) | Space/Materials | 20.90 | 20.90 |
|  | GeneXpert capital & overhead costs | Space/Materials | 1.64 | 1.83 |
|  | Laboratory technician | Staff time | 3.65 | 3.65 |
|  | Laboratory visit | Space/Materials | 5.23 | 5.25 |
| Viral load negative result | HCV clinic receptionist | Staff time | 0.25 | 0.25 |
|  | HCV nurse | Staff time | 1.76 | 1.76 |
|  | HCV medical doctor | Staff time | 0.79 | 0.79 |
|  | HCV clinic visit | Staff time | 3.43 | 3.45 |
|  | Patient support nurse | Staff time | 0.85 | 0.85 |
|  | Patient support visit | Space/Materials | 0.95 | 0.95 |
| Viral load positive result | HCV clinic receptionist | Staff time | 0.25 | 0.25 |
|  | HCV nurse | Staff time | 1.76 | 1.76 |
|  | HCV medical doctor | Staff time | 0.79 | 0.79 |
|  | HCV clinic visit | Staff time | 3.43 | 3.45 |
|  | Patient support nurse | Staff time | 1.32 | 1.32 |
|  | Patient support visit | Space/Materials | 0.95 | 0.95 |
|  | Laboratory technician | Staff time | 0.53 | 0.53 |
|  | Laboratory visit | Space/Materials | 5.23 | 5.25 |
| Baseline initial assessment | HCV clinic receptionist | Staff time | 0.25 | 0.25 |
|  | HCV nurse | Staff time | 1.76 | 1.76 |
|  | HCV medical doctor | Staff time | 1.51 | 1.51 |
|  | HCV clinic visit | Space/Materials | 3.43 | 3.45 |
|  | Patient support nurse | Staff time | 0.79 | 0.79 |
|  | Patient support visit | Space/Materials | 0.95 | 0.95 |
| Family planning (maternal and child health clinic-MCH) referral | MCH nurse | Staff time | 1.52 | 1.52 |
|  | MCH visit | Space/Materials | 0.16 | 0.16 |
| Baseline subsequent assessment | HCV clinic receptionist | Staff time | 0.25 | 0.25 |
|  | HCV nurse | Staff time | 1.76 | 1.76 |
|  | HCV medical doctor | Staff time | 0.79 | 0.79 |
|  | HCV clinic visit | Space/Materials | 3.43 | 3.45 |
| Treatment initiation | HCV clinic receptionist | Staff time | 0.25 | 0.25 |
|  | HCV nurse | Staff time | 1.76 | 1.76 |
|  | HCV medical doctor | Staff time | 0.87 | 0.87 |
|  | HCV clinic visit | Space/Materials | 3.43 | 3.45 |
|  | Patient support nurse | Staff time | 0.94 | 0.94 |
|  | Patient support visit | Space/Materials | 0.95 | 0.95 |
| Treatment follow up | HCV clinic receptionist | Staff time | 0.25 | 0.25 |
|  | HCV nurse | Staff time | 1.76 | 1.76 |
|  | HCV medical doctor | Staff time | 0.56 | 0.56 |
|  | HCV clinic visit | Space/Materials | 3.43 | 3.45 |
|  | Laboratory technician | Staff time | 0.40 | 0.40 |
|  | Laboratory visit | Space/Materials | 5.23 | 5.25 |
| On treatment counselling | Patient support nurse | Staff time | 0.71 | 0.71 |
|  | Patient support visit | Space/Materials | 0.95 | 0.95 |
| End of treatment | HCV clinic receptionist | Staff time | 0.25 | 0.25 |
|  | HCV nurse | Staff time | 1.76 | 1.76 |
|  | HCV medical doctor | Staff time | 0.60 | 0.60 |
|  | HCV clinic visit | Space/Materials | 3.43 | 3.45 |
|  | Laboratory technician | Staff time | 0.53 | 0.53 |
|  | Laboratory visit | Space/Materials | 5.23 | 5.25 |
|  | Patient support nurse | Staff time | 0.71 | 0.71 |
|  | Patient support visit | Space/Materials | 0.95 | 0.95 |
| SVR12 | HCV clinic receptionist | Staff time | 0.25 | 0.25 |
|  | HCV nurse | Staff time | 1.76 | 1.76 |
|  | HCV medical doctor | Staff time | 0.80 | 0.80 |
|  | HCV clinic visit | Space/Materials | 3.43 | 3.45 |
|  | Laboratory technician | Staff time | 0.53 | 0.53 |
|  | Laboratory visit | Space/Materials | 5.23 | 5.25 |
|  | Patient support nurse | Staff time | 0.74 | 0.74 |
|  | Patient support visit | Space/Materials | 0.95 | 0.95 |

## Table S8. Unit costs for visits, laboratory tests, test kits and medicines

| Item | Resource type | Unit cost (US$) | |
| --- | --- | --- | --- |
|  |  | Financial | Economic |
| **Consultations/ visits** | | | |
| Outpatient department consultation | Visit | 3.37 | 3.37 |
| HCV Rapid Diagnostic (RDT) Test | Visit | 5.63 | 5.65 |
| Viral load negative result | Visit | 8.03 | 8.04 |
| Viral load positive result | Visit | 14.26 | 14.28 |
| Baseline initial assessment | Visit | 8.69 | 8.71 |
| Family planning referral | Visit | 1.69 | 1.69 |
| Baseline subsequent assessment | Visit | 6.23 | 6.24 |
| Treatment initiation | Visit | 8.20 | 8.21 |
| Treatment follow up | Visit | 11.63 | 11.66 |
| On treatment counselling | Visit | 1.65 | 1.65 |
| End of treatment | Visit | 13.46 | 13.48 |
| SVR12 | Visit | 13.69 | 13.72 |
| Coordination and general HCV overhead costs | Per patient diagnosed | 69.79 | 69.90 |
| **Laboratory tests, test kits and medicines** | | | |
| Albumin | Laboratory test | 0.12 | 0.12 |
| Alkaline phosphatase | Laboratory test | 1.20 | 1.20 |
| Anti HEV | Laboratory test | 6.88 | 6.88 |
| Bilirubin - Total & Direct | Laboratory test | 1.12 | 1.12 |
| Calcium | Laboratory test | 1.29 | 1.29 |
| Complete blood count & Erythrocyte sedimentation rate | Laboratory test | 3.44 | 3.44 |
| Complete blood count | Laboratory test | 2.15 | 2.15 |
| Creatinine | Laboratory test | 1.20 | 1.20 |
| Glucose | Laboratory test | 1.03 | 1.03 |
| Hepatitis surface antigen test (HBsAg) | Laboratory test | 1.15 | 1.15 |
| HCV Genotype | Laboratory test | 64.52 | 64.52 |
| Oraquick - HCV RDT | Laboratory test | 8.02 | 8.02 |
| GeneXpert - HCV RNA | Laboratory test | 24.09 | 24.09 |
| Haemoglobin | Laboratory test | 1.29 | 1.29 |
| HIV RDT | Laboratory test | 0.93 | 0.93 |
| Pregnancy test | Laboratory test | 0.19 | 0.19 |
| Prothrombin /International Normalized Ratio (PT/INR) | Laboratory test | 1.89 | 1.89 |
| ALT (SGPT) | Laboratory test | 1.29 | 1.29 |
| AST (SGOT) | Laboratory test | 1.29 | 1.29 |
| Sputum acid-fast bacilli (Routine) | Laboratory test | 1.29 | 1.29 |
| Thyroid Stimulating Hormone (TSH) | Laboratory test | 10.32 | 10.32 |
| Syphilis | Laboratory test | 2.58 | 2.58 |
| Daclatasvir 60mg (per tablet) | DAA medicines | 0.76 | 0.76 |
| Sofosbuvir 400mg (per tablet) | DAA medicines | 2.60 | 2.60 |
| Ribavirin 200mg (per tablet) | Medicines | 0.31 | 0.31 |
| Sofosbuvir 400mg-Velpatasvir 100mg (per tablet) | DAA medicines | 3.39 | 3.39 |
| Peginterferon alfa-2a (per week) | Medicines | 61.82 | 61.82 |
| Sofosbuvir 400mg-Ledipasvir 90mg (per tablet) | DAA medicines | 11.41 | 11.41 |
| Ultrasound | Laboratory test | 5.16 | 5.16 |

## Table S9. Variables used in the univariate sensitivity analysis

| Parameter | Base-case value | Sensitivity, Low | Sensitivity, High |
| --- | --- | --- | --- |
| Discount rate (%) | 3 | 0 | 7 |
| Initial cohort age (years) | 41.3 | 25 | 65 |
| Cost of sofosbuvir + daclatasvir for 12 weeks ($US) | $282 | $75 |  |
| DAA treatment coverage (%) | 51.5 |  | 80 |
| SVR rate (%) | 84 |  | 94 |
| HCV antibody prevalence in testing (%) | 33 | 5.5[9] | 80 |
| Time horizon (years) | Lifetime | 20 |  |
| Annual cost of care for liver disease ($USD) | 0 | F0: $21  F1: $22  F2: $31  F3: $56  F4: $50  DC: $278  HCC: $339 |  |
| Reinfection rate (per 1000 person years) | 0 |  | 3.7[9] |

## Table S10. Proportion of total costs per patient diagnosed, treated, or cured attributable to components of treatment costs, Mean (SD)

| Outcome | Clinic visits | Lab tests | DAA medicines | Diagnosis | Total Cost |
| --- | --- | --- | --- | --- | --- |
| Diagnosed (n=4764) | $120.43  ($47.90) | $78.17  ($78.04) | $244.62  ($283.01) | $122.95  ($0) | $546.17  ($387.94) |
| Average % of total | 22% | 14% | 45% | 23% |  |
| Initiated treatment (n=2453) | $159.87  ($34.33) | $120.75  ($83.24) | $436.24  ($251.45) | $122.95  ($0) | $839.81  ($335.86) |
| Average % of total | 19% | 14% | 52% | 15% |  |
| Cured (n=1641) | $171.08  ($28.43) | $150.27  ($72.06) | $496.74  ($243.61) | $122.95  ($0) | $941.04  ($312.08) |
| Average % of total | 18% | 16% | 53% | 13% |  |

# Supplementary Figures

## Figure S1: Diagnostic and treatment algorithm for patients in Hepatitis C treatment program.

Screening risk factors:(1) Symptomatic patient (2) Intravenous drug use (3) HIV positive (4) Spouse of HCV-positive patient (5) Child of HCV-positive mother (6) Mother of HCV-positive child (7) History of incarceration (8) History of jaundice (9) History of blood transfusion (10) Major surgeries/dental procedures (11) Injections/infusions at local clinics (12) Others (dialysis history, history of minimal invasive procedures, health care personnel, Men who have sex with men (MSM)). OPD = outpatient department, PS = patient support.


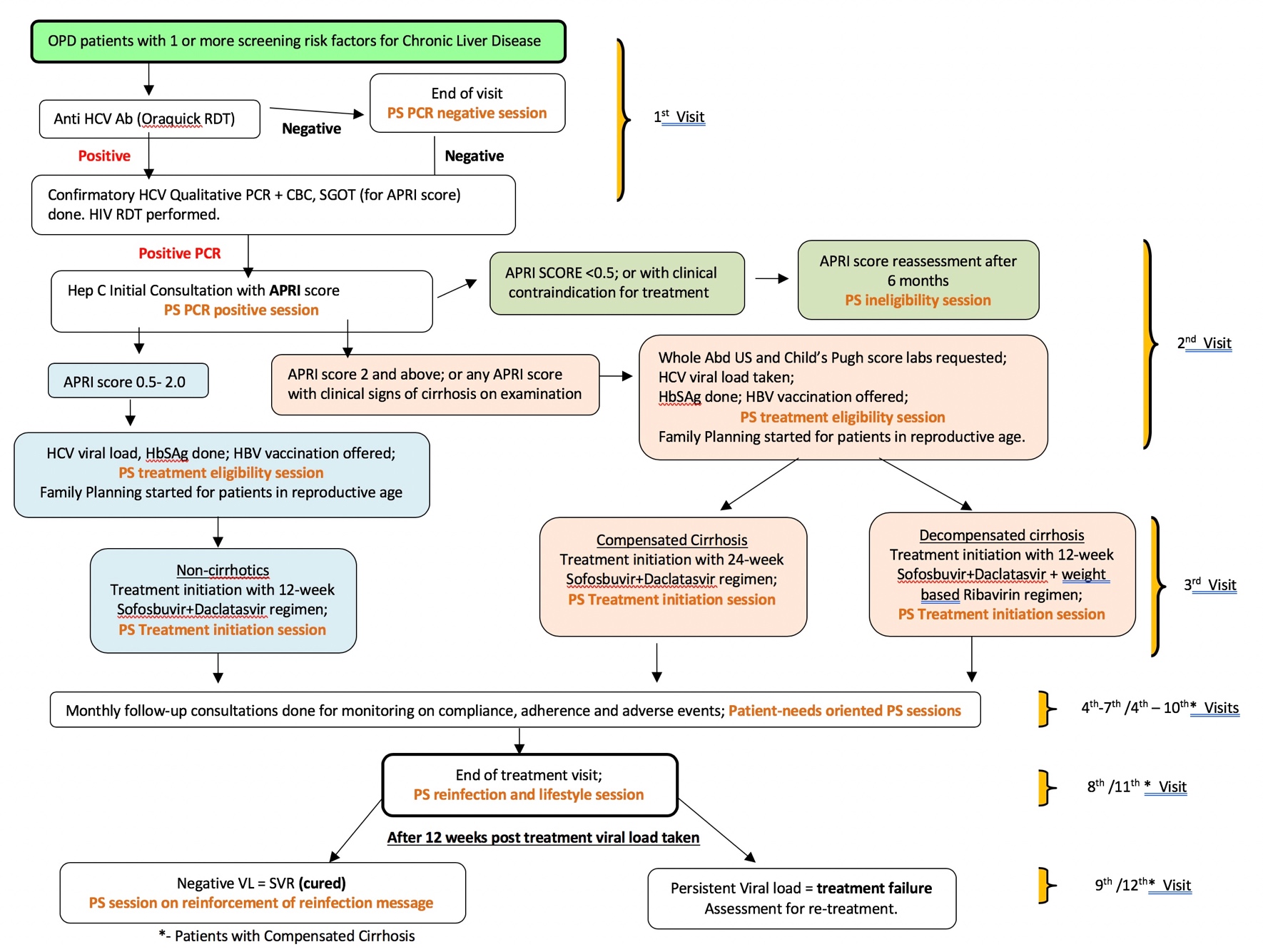


## Figure S2: Cost-effectiveness plane

Cost-effectiveness plane showing the difference in costs and quality adjusted life years per chronically infected individual for 1,000 simulations. DALY – disability adjusted life year. Lines show the willingness to pay threshold based on GDP per capita in Pakistan (solid line), or an alternative recently published threshold intended to capture health opportunity costs in Pakistan [11] (dashed line).

## Figure S3: Cost-effectiveness acceptability curve

Cost-effectiveness acceptability curve for direct-acting antiviral-based HCV treatment in comparison to no treatment. DALY – disability adjusted life year. Lines show the willingness to pay threshold based on GDP per capita in Pakistan (solid line), or an alternative recently published threshold intended to capture health opportunity costs in Pakistan [11] (dashed line).

# References

1. Harris, P.A., et al., *Research electronic data capture (REDCap)—A metadata-driven methodology and workflow process for providing translational research informatics support.* Journal of Biomedical Informatics, 2009. **42**(2): p. 377-381.

2. Walker, J.G., et al., *Simplifying the HCV care model to scale up HCV treatment in Cambodia: an economic evaluation*.

3. World Health Organisation. *CHOosing Interventions that are Cost Effective (WHO-CHOICE). Health service delivery costs. Estimates of Unit Costs for Patient Services for Pakistan.* 2005 14/11/2018]; Available from: <http://www.who.int/choice/country/pak/cost/en/>.

4. Morgan, R.L., et al., *Eradication of hepatitis C virus infection and the development of hepatocellular carcinoma: a meta-analysis of observational studies.* Ann Intern Med, 2013. **158**(5 Pt 1): p. 329-37.

5. Thein, H.H., et al., *Estimation of stage-specific fibrosis progression rates in chronic hepatitis C virus infection: a meta-analysis and meta-regression.* Hepatology, 2008. **48**(2): p. 418-31.

6. van der Meer, A.J., et al., *Association between sustained virological response and all-cause mortality among patients with chronic hepatitis C and advanced hepatic fibrosis.* Jama, 2012. **308**(24): p. 2584-93.

7. Attaullah, S., S. Khan, and I. Ali, *Hepatitis C virus genotypes in Pakistan: a systemic review.* Virology Journal, 2011. **8**(1): p. 433.

8. Kanwal, F., et al., *HCV genotype 3 is associated with an increased risk of cirrhosis and hepatocellular cancer in a national sample of U.S. Veterans with HCV.* Hepatology, 2014. **60**(1): p. 98-105.

9. Lim, A.G., et al., *Curbing the hepatitis C virus epidemic in Pakistan: the impact of scaling up treatment and prevention for achieving elimination.* Int J Epidemiol, 2018. **47**(2): p. 550-560.

10. Holmberg, S.D., et al., *Noninvasive serum fibrosis markers for screening and staging chronic hepatitis C virus patients in a large US cohort.* Clin Infect Dis, 2013. **57**(2): p. 240-6.

11. Ochalek, J., J. Lomas, and K. Claxton, *Estimating health opportunity costs in low-income and middle-income countries: a novel approach and evidence from cross-country data.* BMJ Glob Health, 2018. **3**(6): p. e000964.
